# Trial femoral head loss in to the soft tissues of pelvis during primary total hip replacement: a case report

**DOI:** 10.1186/1757-1626-1-151

**Published:** 2008-09-12

**Authors:** Arunas Vertelis, Linas Vertelis, Sarunas Tarasevicius

**Affiliations:** 1 Department of Orthopedics, Kaunas Medical University, Kaunas, Lithuania

## Abstract

The loss of trial femoral head in the soft tissues is a rare per operative complication in total hips replacement. We report the loss of the femoral head in surrounding hip joint soft tissues and unsuccessful attempts to locate and remove it. Surgeons should be aware of such complication as trial femoral heads usually are made from non radiolucent material and cannot be detected by regular x-ray examination during surgery. The industry should consider manufacturing trial femoral heads from x-ray visible material.

## Case report

**Figure 1:**
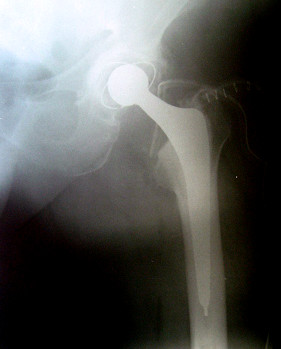
X-ray examination of total hip replacement patient during surgery after loss of femoral head.

76 years old lady with severe osteoarthritis was admitted for elective total hip replacement at our institution. She was operated through the posterior approach under spinal anesthesia. All polyethylene acetabular cup and cemented femoral stem were inserted. Afterwards the trial, 28 mm diameter femoral head was mounted on the femoral neck of the stem. The hip joint was relocated and the stability, range of motions, risk of dislocations was examined. During the following hip dislocation the trial femoral head was lost in surrounding soft tissues. The revision of soft tissues was performed and 3 – 3.5 cm defect of anterior joint capsule was observed. The trial head was found in this particular anterior capsule defect, in approximately 4 – 5 cm of depth. Attempts to remove the head with fingers were unsuccessful. Attempt to grip the head with Kocher clamp was unsuccessful too and subsequently the head disappeared. All further attempts to find it were not successful. After numerous attempts the operation was finished in usual way. After closing the wound the radiographic examination of the prosthetic hip joint was performed but now signs of lost femoral head were observed (Figure 1).

**Figure 2:**
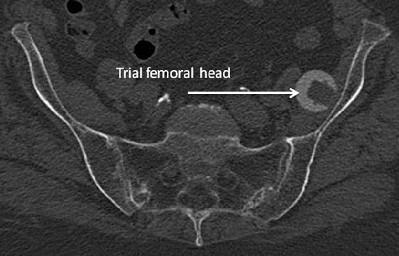
CT-Scan of prosthetic patient with lost femoral head, 5 days after surgery.

Post-operative period was usual. Patient started to walk the next day using crutches, with full weight bearing. The femoral head was located with computed tomography (CT) performed on fifth post-operative day. The head was in sacroiliac arteries zone, between hip bone and illiopsoas muscle (Figure 2).

At 3 months follow up patients prosthetic hip was asymptomatic. Eight months after the surgery additional CT scan was performed. The head was found in the same place as in previous CT and no reaction of surrounding tissues was observed.

## Discussion

We found very few reports in the literature describing similar cares. O. Batouk and M. Gilbart [1] described the intraoperative loss of trial femoral head and unsuccessful attempts to remove it. A. Princep et al. [2] described the options how to remove the migrated femoral head. He suggested that pressing the supra-ingvinal area, also flexing the hip would be helpful. A tea-spoon, as a tool was suggested for removal of the trial femoral head by the author. D. Alfonso et al. [3] suggested fixing the trial head with a strong suture to prevent its loss.

We observed no complications related to the femoral head loss in pelvis in our case. However the prevention of such events is of importance. Despite the all precautions these events might happen and orthopedics surgeon should have tools resolve this problem. The trial femoral head is not notable on x-rays and the CT scan during the prosthetic surgery is complicated. The industry should consider manufacturing trial femoral heads from the material which could be visible during conventional x-ray examination.

## Consent

Written informed consent was obtained from the patient for publication of this case report and accompanying images. A copy of the written consent is available for review by the Editor-in-Chief of this journal.

## Competing interests

The authors declare that they have no competing interests.

## Authors' contributions

AV performed the surgery, wrote the manuscript. LV performed literature analysis, edited the manuscript. ST organized the preparation of case report, edited the manuscript.
